# Response to the letter to the Editor “Regional citrate anticoagulation for intermittent hemodialysis in the intensive care. What is the optimal set-up?” by Gubensek et al.

**DOI:** 10.1186/s13613-021-00859-9

**Published:** 2021-05-03

**Authors:** Christophe Leroy, Alexandre Lautrette

**Affiliations:** 1 Medical Intensive Care Unit, Gabriel-Montpied University Hospital, Clermont-Ferrand, France; 2 Intensive Care Unit, Regional Hospital Center, Puy en Velay, France; 3 LMGE (Laboratoire Micro-Organismes: Génome et Environnement), UMR CNRS 6023, Université Clermont Auvergne, Clermont-Ferrand, France; 4 Intensive Care Unit, Centre Jean Perrin, 54 Rue Montalembert, BP69, 63003 Clermont-Ferrand, Cedex 1, France

We read with interest the letter by Gubensek et al. [1] on our article recently published in Annals of Intensive Care [2] comparing regional citrate anticoagulation (RCA) and heparin for intermittent hemodialysis in ICU patients. In their letter, the authors comment on the two major findings of our study: the safety and the high rate of premature termination, which could be associated with the RCA protocol we used. They propose another RCA protocol using a citrate infusion in combination with a calcium-free dialysate.

Optimal anticoagulation is the cornerstone of successful renal replacement therapy (RRT). The intensivist must adjust the intensity of anticoagulation to prevent premature circuit clotting and the systemic risks of anticoagulation: bleeding for heparin, or cardiac events related to hypocalcemia and citrate accumulation for anticoagulation using the citrate. Gubensek et al. suggest that the safety of the RCA protocol used in our study is questionable, because an additional calcium infusion for hypocalcemia (< 0.9 mmol/L) was performed in 10 sessions (8.4%) in 8 patients. The metabolic disorders associated with RCA are common during intermittent RRT or continuous RRT. In a recent trial on the effect of RCA versus heparin anticoagulation during continuous RRT, Zarbock et al. reported severe hypocalcemia, severe cardiac rhythm disorders, and citrate accumulation in 1.4%, 3.4%, and 0.7%, respectively, of the 300 patients enrolled in the RCA group [3]. Robert et al. reported hypercalcemia in 27.6% of intermittent RRT sessions in which anticoagulation with a calcium- and magnesium-free citrate-containing dialysate was used [4]. In our study, we observed no cardiac events or citrate accumulation. We maintain that the clinical safety of the RCA protocol we used is at least as acceptable as that reported in the literature. We agree with Gubensek et al. that close monitoring of ionized calcium during intermittent RRT or continuous RRT remains mandatory to treat metabolic abnormalities before a clinical event.

As pointed out by Gubensek et al., the high rate of premature termination in the RCA group could have been due to the use of calcium-containing dialysate, as observed in their study of a small cohort of chronic hemodialysis patients [5]. The use of a calcium-free dialysate could be an interesting technique for regional anticoagulation, as reported elsewhere [4, 6]. We agree with Gubensek et al. that effective anticoagulation is mainly achieved by calcium loss through the filter and that monitoring of ionized calcium should be recommended to prevent clinical events, because the occurrence of metabolic abnormality during this procedure is always possible. Gubensek et al. propose anticoagulation using a citrate infusion in combination with a calcium-free dialysate. It is true that this association could greatly decrease the high rate of premature filter loss due to clotting. However, this protocol could result in major and rapid calcium loss, which contributes effectively to clotting prevention but also carries the risk associated with the hypocalcemia. The elevated rates of blood flow and dialysate used in intermittent RRT increase the risk of sudden and fatal cardiac events due to hypocalcemia caused by human error or problems with calcium infusion, especially in ICU patients, in whom hemodynamic instability is frequent. The authors of the letter state that RCA using a citrate infusion with a calcium-free dialysate for intermittent RRT is routinely performed in their ICU. However, there is as yet no documented assessment of their procedure in an ICU cohort. It seems reasonable, therefore, to suggest that the safety and efficacy of this procedure using two means of calcium loss (citrate and calcium-free dialysate) should be assessed in scientific reports published in peer-reviewed journals before it is adopted for daily clinical practice in an ICU population. Assistance from the manufacturers of RRT would be required to develop an automated protocol with adjusted citrate and calcium infusions to minimize risks and simplify its use in all ICUs, as has been done for the continuous RRT.

## Data Availability

Not applicable.

## Abbreviations

RRT: Renal replacement therapy
ICU: Intensive care unit
RCA: Regional citrate anticoagulation
